# Sleep waves and recovery from drug and alcohol dependence: Towards a rhythm analysis of sleep in residential treatment

**DOI:** 10.1016/j.socscimed.2017.05.016

**Published:** 2017-07

**Authors:** Robert Meadows, Sarah Nettleton, Joanne Neale

**Affiliations:** a Department of Sociology, University of Surrey, Guildford, Surrey, GU2 7XH, United Kingdom; b Department of Sociology, University of York, Heslington, YO10 5DD, United Kingdom; c National Addiction Centre, 4 Windsor Walk, Institute of Psychiatry, Psychology & Neuroscience, King's College London, Denmark Hill, London, SE5 8BB, United Kingdom

**Keywords:** United Kingdom, Sleep, Drug and alcohol dependence, Rhythmanalysis

## Abstract

This paper reports on a study of sleep amongst men and women who are living in residential rehabilitation centres in the UK and who are receiving support for their recovery from addiction to alcohol and other forms of substance use. Conceptually and methodologically, the paper draws on the work of the French sociologist Lefebvre and, in particular, his rhythmanalysis. We argue that this approach offers a useful way of exploring sleep in terms of biological, experiential, temporal, spatial and social rhythms. It also has the potential to facilitate interdisciplinary dialogue. Empirical data comprising qualitative interviews with 28 individuals, sleep diaries, and actigraphy reports (which measure movement as a proxy for sleep) are examined in combination to generate insights into the challenges associated with sleep in recovery from substance misuse. We examine how sleep in recovery involves an alignment of the spatiotemporal rhythms of rehabilitation and the multiple embodied rhythms of individuals. Institutionalised routines reproduce and impose ideas of day/night sleep cycles which are presumed to accord with ‘natural’ circadian rhythms. Although study participants very much want to achieve these ‘natural hegemonies’ of sleep, alignment of individual and institutional rhythms is difficult to achieve. We develop the notion of ‘*sleep waves*’ as an analytic to capture the multifaceted elements of sleep and to argue that sleep waves recur but are also shaped by complex networks of rhythms, rituals and routines. *Sleep waves* can become relatively stabilised in rehabilitation settings, but the anticipation of moving on disturbs rhythms and generates anxieties which can affect recovery.

## Highlights

• Provides a sociological perspective on sleep, drug and alcohol dependence and recovery.
• Introduces *sleep waves* as an analytic to bring multiple aspects of sleep together.
• Draws on an innovative mix of interview and movement data to explore *sleep waves.*
• Argues that residential rehabilitation (re)enforces ‘natural hegemonies’ of sleep.
• Opens up a space for interdisciplinary dialogue and interventions.

## 1 Introduction

The paper reports on a study of the sleep of men and women living in drug and alcohol residential treatment services. Prompted by a biomedical literature that indicates that good sleep can play a critical role in recovery (Conroy and Arnedt, 2014) and a separate sociological literature that indicates that in residential treatment sleep, subjectively at least, can improve (Nettleton et al., 2011a, Neale et al., 2012), the aim of this article is to deploy Lefebvre's (2004 [1992]) *rhythmanalysis* to make sense of empirical data on sleep in these settings. Lefebvre's rhythmanalysis, at once a conceptual and a methodological approach, is apposite to sleep because it seeks to examine the temporal, material and relational aspects of embodied social life. The central concept - *rhythm* - takes many interconnected forms. Embodied rhythms are, for instance, related to spatial rhythms, temporal rhythms, natural rhythms, and cosmic rhythms (Rantala and Valtonen, 2014, Schwanen et al., 2012). This focus on temporalities and rhythms finds resonance in biomedical literature on sleep in general, and sleep and drug use in particular, indicating potential for interdisciplinary research on this topic. The lexical affinities of socio-biological clocks and rhythms, although rooted in divergent epistemological traditions, offer opportunities for dialogue across the human sciences. Rhythmanalysis may therefore be a way to respond to calls within the sociology of health and illness for interdisciplinary exchange between social and natural scientists (Rose, 2013, Timmermans and Haas, 2008).

We foreground our empirical data with a brief review of how sleep is described in temporal and rhythmic terms within the natural and social science disciplines. We then offer a summary of Lefebvre's rhythmanalysis and his use of the wave metaphor. We find that across disciplines there is an emphasis on the play between multiple endogenous (internal) and exogenous (external) rhythms indicating scope for the analysis of sleep as embodied: viscerally and socially. These analytical insights give purchase on the interpretation of our empirical data, which comprise qualitative interviews, self-completion sleep diaries, and actigraphs. Throughout our analysis we play with the idea of *sleep waves* as a foil to the neurophysiological articulation of the term as used in the vocabulary of ‘circadian rhythms’ and ‘slow wave sleep’. We introduce these terms that are core to sleep science and then turn to Lefebvre's conceptualisation of rhythms and waves, and suggest that a sociological notion of s*leep waves* serves to capture the extent to which sleep is repetitive and rhythmic and always a combination of biological and social contingencies.

## 2 A language of rhythms: the circadian and entrainment

Sleep scientists work on the premise that sleep combines two interrelated processes: sleep pressure (a homeostatic process) which increases as individuals remain awake and decreases as they sleep, and circadian rhythm (the ‘internal biological clock’) which is unaffected by sleep deprivation (Borbély, 1982). They assert that the homeostatic and circadian processes are interlinked: homeostatic processes primarily determine ‘slow-wave sleep’, whilst the circadian rhythm regulates Rapid Eye Movement (REM) sleep (Dijk and von Schantz, 2005, Arnedt et al., 2012). In ‘healthy’ sleep, the endogenous circadian clock is aligned with, or in the language of sleep science ‘*entrained’* by, diurnal cues, known as ‘*zeitgebers’,* such that ‘normal’ sleep is in tune with the day/night cycle (Dijk and Lazar, 2012). Light is considered the dominant stimulus for this ‘entraining’ of circadian rhythms to local temporal environments (Mistlberger and Skene, 2004). Sleep science, then, frames sleep as a series of chronobiologically endogenous processes and circadian rhythms which are nevertheless influenced by external social factors and social ‘clocks’.

Research on sleep and addiction currently prioritises a focus on endogenous (internal) processes; exploring alterations in circadian systems with exposure to substances of abuse (Arnedt et al., 2012, Falcón and McClung, 2009). For example, studies of alcohol-dependent adults at two weeks into withdrawal show phase differences in melatonin profiles relative to ‘healthy’ controls (Hasler et al., 2012). Additionally, male heroin-dependent individuals show disruption in cortisol rhythms three days post cessation, but not by day ten, suggesting that the first few weeks of abstinence may be a key time for chronobiologically informed treatments. However, the same individuals also show longer-term disruption to the rhythms of the ‘clock’ genes (identified as PER1 and PER2). These genes are also implicated in reward processing, with clinical scientists suggesting that their continued disruption may contribute to persistent craving and withdrawal (Hasler et al., 2012, Hasler et al., 2014).

Despite this focus on endogenous processes, the model of sleep underpinning this research remains one which talks of both internal and external rhythms. The study of chronobiology can be linked to ‘chronogeography’, wherein social places are continually ‘(re)made through the intersection of multiple rhythms’ (Lager et al., 2016: 4). Places are understood to be generative of rhythmic events (such as, job shifts, opening hours of shops, transport timetables, festivals and so on) which are said to act like ‘pacemakers’ (or *zeitgebers* see above), pacing both single behaviours and constellations of behaviours (Parkes and Thrift, 1979: 361). ‘Pacemakers’ of the night-time economy, for example, may include opening hours and availability of nightlife facilities, perceptions of crime, disorderliness and safety, and availability of transport (Parkes and Thrift, 1979: 362). In combination, studies point to the interconnectedness of socio-temporal, spatial, experiential and biological rhythms, suggesting that Lefebvre's rhythmanalysis may be a particularly apposite analytic for comprehending sleep within given socio-spatiotemporal configurations. Lager et al. (2016) suggest that Lefebvre's *rhythmanalysis* links chronobiology with chronogeography, highlighting how the ‘rhythmic orderings of people and place come into being and inform their experiences’ (p.1565). As we have seen, the life sciences focus on the endogenous but also acknowledge – although they do not elaborate on - the exogenous. So rhythmanalysis offers a tool to integrate disciplinary approaches.

## 3 Rhythms, waves, everyday life and sleep

Lefebvre's (2004) *Rhythmanalysis* is primarily an approach that seeks to capture the interplay of multiple rhythms – biological, experiential, spatial, temporal and social. A rhythmanalysis involves an ‘analytic operation’ to identify ‘the plurality of rhythmic interactions’ which Lefebvre refers to as ‘polyrhythms’ (2004:42). It is crucial here to appreciate how he conceives rhythm; specifically, it involves repetition, but unlike the mechanical repetitious thud of machines, embodied and social repetition or rhythms *never* replicate their repetition, instead they invariably generate ‘something new and unforeseen’ (2004:8). Lefebvre most effectively communicates this idea of rhythm through the use of a maritime metaphor.

‘To grasp rhythm and polyrhythmias in a sensible, preconceptual but vivid way, it is enough to look carefully at the surface of the sea. Waves come in succession: they take shape in the vicinity of the beach, the cliff, the banks. The waves have a rhythm, which depends on the season, the water and the winds, but also on the sea that carries them, that brings them. […] But look closely at each wave. It changes ceaselessly.’ (2004: 79)

The wave indicates incessant repetition yet with constant change as the interconnections of a multitude of things, objects, atmospheres, and processes create each wave as a unique configuration. Each sea has its rhythm, yet if we ‘look closely at each wave’ we might begin to grasp how bundles of movements, spaces, and objects alter’ (p79). Transferring the maritime metaphor to social contexts, he advises that we examine spaces, objects, people and movements as they combine to generate rhythms in all their uniqueness.

This metaphor is important for our argument because it foregrounds the interconnectedness and contingent nature of rhythms. We carry the metaphor through to the polyrhythmias of sleep, enabling us to think in terms of *sleep waves.* Sleep waves come in succession and take shape according to their vicinity. The notion of *sleep waves* allows us to capture sleep as repetitious, but crucially also rhythmic, thus recognizing that each and every experience of sleep is unique. In sum, human sleep is experientially distinctive *and* yet it is also fundamentally shaped by spatial, temporal, physical and social contexts. *Sleep waves* captures the multiple rhythms of sleep.

*Sleep waves* as an analytic therefore prompt us to (i) capture the rhythm and repetitions of rhythms over time and space; (ii) explore how these everyday rhythms come into being by situating them against aggregate and experiential rhythms in place and the pacemakers of multiple territories; (iii) explore how this rhythmic ordering *affects* individuals' sense of sleep (cf. Lager et al., 2016). We begin to address these issues in our empirical work which comprises subjective accounts and (proxy) measures of the rest-activity circadian rhythm.

## 4 Study design and method

The empirical data presented in this article were generated through a study of 28 men and women who were in receipt of support for their recovery from addiction to alcohol and/or other drugs in England. At the time of the study, they were living in one of two residential rehabilitation services. Centre one provided support for men and women and offered supervised detoxification, which lasted four to six weeks, followed by a main treatment programme comprising group therapy, one-to-one counselling, creative workshops, complementary therapies, and participation in household duties. Days and nights were highly structured, with regular times for waking, meals, classes, bed, and sleep. Each day began with a collective meeting and, when residents left, if successfully completing their treatment, there was a formal gathering to acknowledge their achievements. Centre Two provided support only for women and involved a less structured programme. Residents shared daily activities, such as shopping, cooking, eating and budgeting and, as their recoveries progressed, were permitted to come and go from the service and have overnight stays at home. Bedtime rules were more flexible than in Centre One, although there was still a requirement that residents be in bed at night and up during the day. In both centres, all residents shared bedrooms, and total abstinence was required, although smoking tobacco was permitted.

These particular settings provide a pertinent example of those spaces that Lefebvre describes as being akin to the ‘cloister’, where ‘real life’ is suspended and contemplation encouraged (1991:94). Moreover, Lefebvre advises that methodologically ‘in order to grasp and analyse rhythms, it is necessary to get outside them, but not completely’ (2004: 37). The rhythmanalyst has to find a means to listen and look for patterns, and to this end, our empirical data comprised interviews, self-completion diaries and actigraphy records.

### 4.1 Study sample and data collection techniques

Two of the authors visited the rehabilitation centres and presented the aims of the study to the residents. Potential participants were given written and verbal information about the study including the length and nature of commitment required, and the contact details of the investigators. Prior to participating in the research, all individuals were given the opportunity to ask questions and reflect on their involvement. All participants signed a consent form that clarified that their participation was voluntary, confidential and independent of any professional support they may have been receiving. Twenty eight individuals were interviewed (19 women and 9 men), 25 returned actigraphy data and 19 completed diaries. The youngest was aged 24 and the oldest was 83 years. Seven had been educated to degree level, although most had left school at 16, some with few, if any, qualifications. All had previously received support from either residential and/or community services for their addiction.

**Fig. 1:**
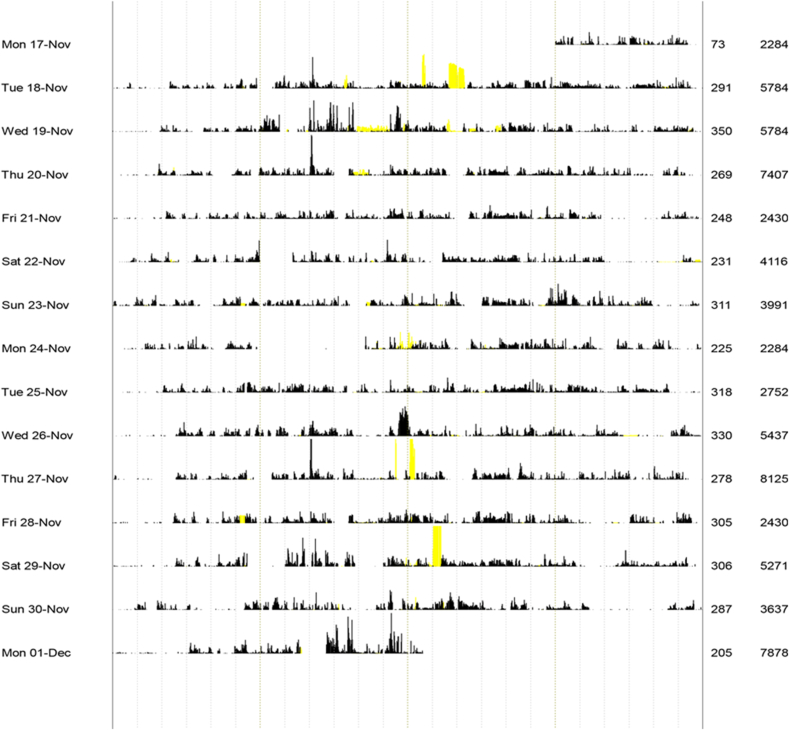
Example actogram.

Participants were requested to wear an actiwatch during the two weeks prior to a qualitative interview and to record their sleep in a structured sleep diary during that same period. An actiwatch is a small, wrist worn, device which measures movement on a minute-by-minute basis. It has been used widely within clinical sleep research as a proxy measure of sleep/wake (Martin and Hakim, 2011) and has been shown to be suitable (Sadeh, 2011) for identifying rhythms. The raw data from the actiwatch can be plotted to create a personalized actogram [Fig. 1]. Each horizontal line in the actogram represents a 24-h period (from midnight to midnight). Black vertical lines represent movement, with the height of the line indicating intensity of movement. In a ‘normal’, monophasic pattern we would expect movement to be concentrated in the middle of each day with little movement to the far right and far left of each line. In Fig. 1, which represents the total two-week period from a single participant, what is immediately of interest is (i) the constant and continuous movement across the 24-h period and (ii) a lack of any clear demarcation between day/night movement. The actigraphy data were downloaded prior to the interviews and used during them to encourage participants to reflect on and interpret their personalized graph. The self-completion diaries (when completed) were also used during the interviews as prompts to recall and to help participants ‘make sense’ of the actigraphy data. The actigraphs and diaries in effect acted as what Latour (1987) would call ‘inscription devices’; encouraging a richer understanding of sleep as they provoked participants to reflect subjectively on seemingly objective measures of movement, rest and sleep.

As well as prompting individuals to reflect on the actigraphy and diary data, the interviews explored participants' biographies, substance use, experiences of rehabilitation, and accounts of their sleep throughout their life course. Particular attention was given to their sleep during the two weeks prior to the interview when most had been wearing the actiwatch. All interviews were audio recorded and transcribed, and ethical approval was secured from Universities of Surrey and York. Names of all participants have been changed for anonymity. Participant identifiers were used to link the interview, diary and actigraphy data, with labels ‘C1’ and ‘C2’ indicating whether participants were interviewed in Centre One or Two respectively.

Interview and actigraphy/diary data were initially analysed independently. Meadows used the raw data from the actiwatch to create non-parametric circadian rhythm variables. These included: i) the ‘interdaily stability’ (IS; a single summary score of the day-to-day variation between the rest-activity rhythm) and ii) the ‘intradaily variability’ (IV; a single summary score of sleep disruption, such as the occurrence of daytime napping and/or night-time arousals over the two-week period). Scores of 1 and 0 are considered ‘perfect’ for IS and IV respectively (Van Someren et al., 1999). Diary data assisted in the identification of periods when the watch was removed (missing data). A graph was then created which plotted each individual's IS against their IV to give an indication of their circadian temporality across the two weeks and in relation to others. Nettleton coded the data using Atlas.ti version 7. Transcripts were also read by the wider team looking for, ‘interferences between linear and cyclical time’ while being sensitive to ‘ambience, tempo and repetitions present in the empirical materials’ (Rantala and Valtonen, 2014: 22). This analysis of rhythms helped to forge *sleep wave* as an emergent concept. Team members discussed the findings emerging from the interview data alongside the actigraphs in order to generate clearer insights into the patterns and variations in the IS and IV plots (Moran-Ellis et al., 2006).

## 5 The routine and rhthyms of residential rehabilition

In his classic text *Hidden Rhythms,* Zerubavel (1981) argues that the temporal profile of each day comprises several components such as: structures, sequences, durations, and routines. The temporal profile of the days and nights were very evident in the two residential rehabilitation centres, and our participants were very aware of them. Harry, for example, articulates the elements of the daily temporalities in Centre One as follows.

‘I'd wake up at six, and then I'd go downstairs. This is when I smoked, and had a cigarette. Then I'd go, half six, go to bed, have my antidepressant, and then go have a coffee or tea, and at quarter to eight […], quarter past eight, breakfast. At the moment, I'm just doing the big wash up after breakfast… and that only takes 20 minutes. And then there's like half an hour with nothing to do, and then at quarter to ten we usually have group psychotherapy, that's an hour to quarter past 11. Then it's usually a lull, there's nothing until one o'clock, lunch. And after lunch there's sometimes something on at two or three. There's always a gap between lunch and that, and some afternoons nothing until dinner, at six. There's lots of down time, and then after dinner, three or four times a week, we've got like NA (Narcotics Anonymous meeting), about half seven.’ (Harry CI:002)

As Harry notes, in the centre, every day has a *sequential structure* (things happen, ‘after’ lunch), a *duration* (‘only takes 20 min’), a *temporal location* (‘I'd go downstairs') and a *rate of recurrence* (‘three of four times a week’). Other participants also storied their days in this way revealing the interconnectedness of the formal routines of the Centre and the individual embodied routines. There is a sense of familiarity borne out of the repetition of activities day after day. The participants can describe the sequences because it is what they have grown accustomed to doing during their stay, and it is what they anticipate they will be doing in the coming days; at least while they are in the Centre itself. The familiarity can engender reassurance and their routines begin to take the form of rituals (Crossley, 2004, Simpson, 2008) or ‘habituated actions’ (Shilling, 2008). Moreover, for many, as these routines become ritualised, they are likely to be experienced as the antithesis to their lives outside formal structured and institutional support (Nettleton et al., 2011b).

Residential rehabilitation also imposes a *spatial location*, in addition to the *temporal location* and *sequential structure* of sleep. Sleep should occur at night and in a bedroom; there are rules at work to enforce this spatio-sequential structuring. Deidre (C2:035) alludes to this in response to the researcher's question, ‘Are you allowed to nap or anything…?’:

Deidre: No
Interviewer: No?
Deidre: Not allowed, frowned upon.
Interviewer: Oh, is it?
Deidre: Yeah, not allowed.
Interviewer: Why is it frowned upon?
Deidre: Don't know, you can ask them that, you're the sleep professional, you can ask them. Just say one of the girls said we're not allowed to nap during the day. Is there a particular reason?

Residential treatment, then, engages with sleep indirectly through what sleep scientists would refer to as *entraining* capacities, the alignment of circadian with diurnal ‘zeitgebers’ as the regularised rules and routines act as oscillating pacemakers (Dijk and von Schantz, 2005) and are consistent with Borbély's (1982) two-process model of sleep (as described above). Napping, for example, is prohibited in order to protect the ‘normal’ homeostatic process and the build-up of sleep pressure during the day. Zeitgebers are also controlled to ensure the circadian clock runs as it should, as articulated by all the participants who made reference to ‘bedtime’ and, as Emma put it, ‘lights off at eleven’ (C1:029).

**Fig. 2:**
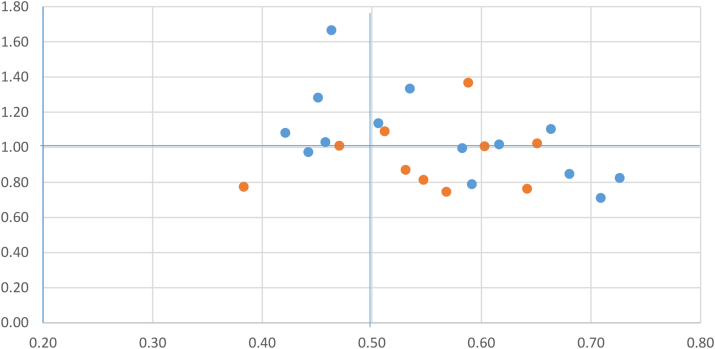
Movements in time and space: Plot of interdaily stability against intradialy variability for participants across both sites.

Nevertheless, the pacemakers and aggregate rhythms of rehabilitation mediate rather than determine entrainment. Fig. 2, ‘*Movements in time and space’*, plots the rest/wake interdaily stability against intradaily variability for participants in both Centres and highlights revealing variations in rest-activity rhythms.

For some residents, we see in Fig. 2 that rest-activity rhythms are rigidly monophasic and repetitive in structure from one day to the next; they are plotted in the bottom right quadrant of Fig. 2. For some, rest-activity rhythms have no clear signs of a monophasic pattern and little consistency across days; they are plotted in the top left quadrant. The individual represented in the actogram above (Fig. 1) is plotted here. For others, rest-activity rhythms have a consistent pattern of being less rigidly monophasic (top right quadrant) or have consolidated sleep periods but at inconsistent times (bottom left quadrant). What these data reveal is variation between participants. While all individuals share the same regularised routines and structures of the treatment centre, their patterns of movement and rest (and in so far as an actigraphy can approximate ‘sleep’) vary. As the Figure shows, there are other individualised rhythms at play. So, for example, those plotted in the top right quadrant are individuals who are routinely inconsistent; in other words, they are *regularly* getting out of bed at the same time each night. The pacemakers which take the form of the ideologies, rules and imposed routines of residential rehabilitation, do not then automatically equate to rhythmic conformity. Entrainment effects are mediated and arbitrated and it is to the more experiential rhythms that we now turn.

## 6 Experiential rhythms

The rationale for the routines of residential treatment is to give shape to the residents' sleep routines. But individual experiences of sleep – *sleep waves* - rely on a bundle of embodied rhythms and polyrhythmias (Lefebvre, 2004). Their *sleep waves* are in part shaped by the rhythms of the institution but also in concert with the aggregation and interaction of their individual experiential rhythms. For some, this interaction can become ‘metonymical’, that is where sleep/wake routines and the routines of rehabilitation become almost synonymous.

### 6.1 ‘Metonymical’ relations

Orla, for example, suggests that structure played an important role in her embodied biography, but it is only now that she can share the institutional logic and appreciate the importance of routine for her sleep. At the time of her interview, Orla was living in Centre Two, but during the interview she elaborated upon her time spent in Centre One, where she had had her ‘first good night's sleep’ for years. Orla goes on to explicitly cite the importance of ‘routine’.

‘Yes, I had four [roommates] all the way through [my residential stay]. That was pretty easy, I was really, really lucky. I had a deaf lady, so there was no chitchat or making noise in the middle of the night… One of the girls… she was very quiet. Yeah, so I was very, very lucky, and there was a curfew there, so you have to be in bed. But again, I think I was getting into a routine, of where I'd been catnapping during the days, because I was too drunk, I was getting into (a) routine of staying awake all day. So by the evenings I was knackered, you know. So it was shifting my routine of not sleeping during the day, so come 11 o'clock, that was it, I was ready for bed.’ (Orla C2:013)

**Fig. 3:**
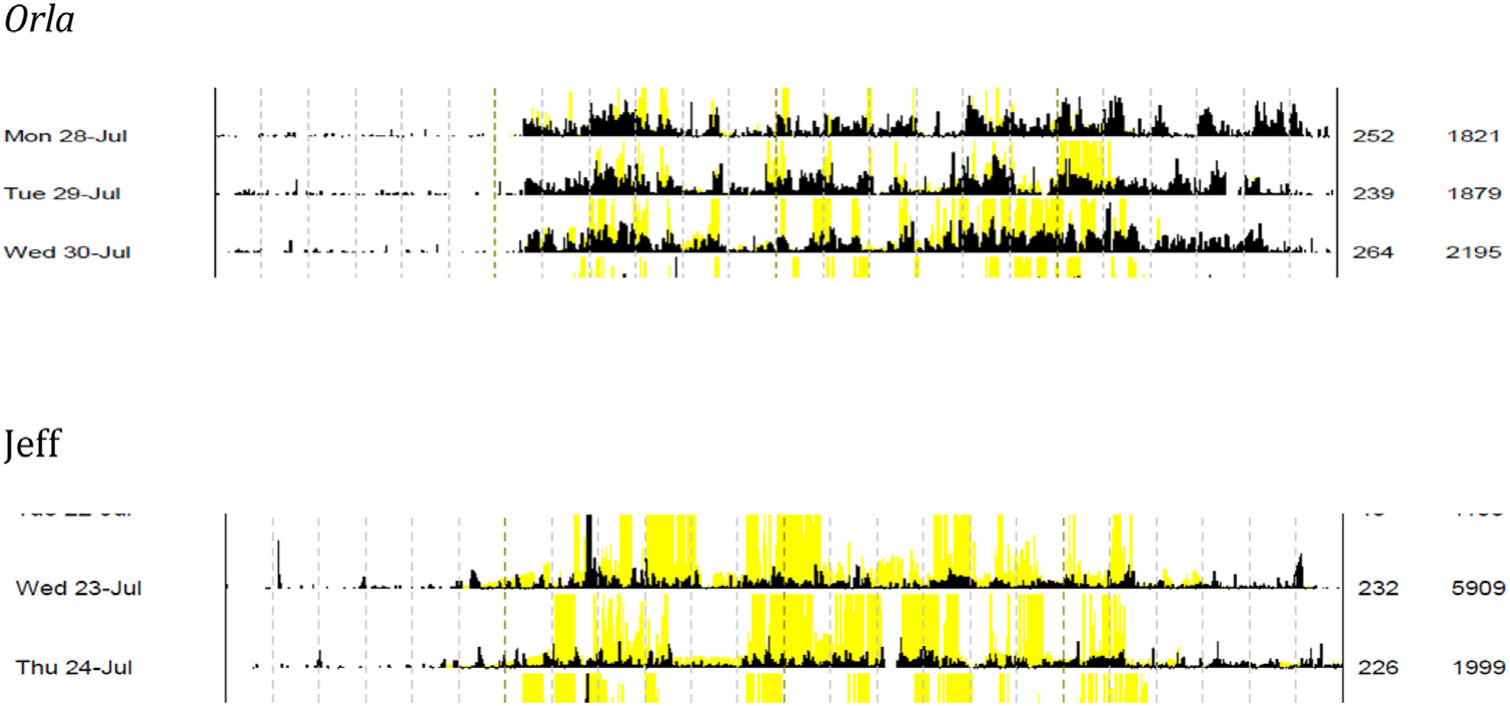
Snapshot of Orla and Jeff.

Orla's narrative portrays a retrospective recognition of the importance of routine and reaches a point where there is a seamless coordination; a process Wolf-Meyer (2011a) describes as ‘metonymical’. Through connection, one entity can be used to refer to another, such as in the join between the spatiotemporal rhythms that both she and the institution see as important. Orla's account also echoes Lefebvre's idea of ‘dressage’; especially when she highlights how the treatment centre regime helped her to ‘get into a routine of staying awake all day’. Through ‘dressage’, Lefebvre (2004) argues, ‘one breaks-in another human living being by making them repeat a certain act, a certain gesture or movement. […] Repetition, perhaps mechanical in (simply behavioural) animals, is ritualised in humans’ (2004: 39). But this is not crude social engineering or biopolitical repression; *rhythmic* transformation of the body can be creative. What is normatively accepted as ‘good’ and ‘healthy’ sleep cannot be willed; it emerges from the repetition of ‘staying awake all day’ and residential treatment becomes the orchestration of a ‘rhythmic intervention’ which shocks the pathologised arrhythmic system back into eurythmia (Jones and Warren, 2016: 288; Meadows, 2012) (see Fig. 3).

We can see from the actigraphy that Orla is located firmly in the bottom right quadrant of Fig. 2 (with an IS of 0.64 and an IV of 0.76). Jeff, interviewed in Centre One, is also located in the same quadrant, but with a less fixed rhythmic pattern (IS of 0.58 and an IV of 0.99); largely, as he explains, because he ‘goes to the toilet about twice a night’. Jeff shows a strong prior and current commitment to sleep/wake routines, and eschews the practice of napping, which for him is an indicator of sleep quality.

‘I have no desire to cat nap at all, which indicates I am happy with my sleep. Whereas in the bad days, let's call them 2000 to 2005, my sleep was intermittent, not regular, and the pattern would look all over the place on the graph which I am looking at’ (Jeff C1:008)

For Orla and Jeff, sleep is not something to be worked on directly; it is created from the routine and is the effect of underlying spatiotemporal rhythms. Aggregate and experiential rhythms become almost synonymous and ‘metonymical’, as captured by a number of participants who recalled that routine was central to their upbringing. This includes Tina who reports: ‘I think I slept very well as a child because I had such good routine’ (C1:004). However, not all participants achieved synchronicity between experiential and the spatial-temporal rhythms, and their embodied sleep was felt to be shaped by alternative or discordant biologies.

### 6.2 Discordant ‘biologies’

Close scrutiny of any social space finds that there are invariably ‘hidden’ routines and rhythms which casual inspection would overlook (Zerubavel, 1981). Residential treatment is no different and we find a hybrid polyrhythmia of intersecting rhythms (Nettleton et al., 2017, forthcoming) and a spatiotemporal landscape that is more complex than institutionalised patterns would suggest. For some residents, a more distinct separation of individual visceral and experiential rhythms and aggregate institutional rhythms persisted. Andrew, for example, is located in the top left quadrant of Fig. 2. He has no clear signs of a monophasic pattern and little consistency across days. Andrew explains how substance use has affected his underlying biological rhythms and that there is no point ‘fighting’ it.

‘Well I think it's just noradrenaline, isn't it? It's just a long-term use of heroin. Like for the first week or two of waking up proper [gasping], like that, you wake up like that, and I thought it was just me, and I saw somebody else do it, and, “you do that as well”? And I spoke to a doctor here who's done some research on it, and it's just a noradrenaline surge. For some reason, because heroin like really increases your ability to sleep, when you take it away, the brain just loses the ability not to produce noradrenaline all the time, it's literally that. And there's no point fighting it, I find. A lot of people [say], ‘oh I have to sleep because it's four in the morning’. Well it's four in the morning, go out and look at the stars. It's not a problem, it really isn't. They toss and turn. No, if I'm alert, I'll go down. Fair enough, if I come back and I get another half hour's sleep in three hours, brilliant, but if I don't…’ (Andrew C1:025)

However, Andrew later explains that he now accepts that if he wants ‘to hold down some kind of fucking life’, his sleep/wake cycles need to align with normative sleep routines. He appears optimistic that this will happen if he stays abstinent as eventually his biological rhythms will bend back to mirror those of ‘normal people’. He comes to internalise normative hegemonic notions of ‘natural’ sleep, what Wolf-Meyer (2011a) refers to as ‘natural hegemonies’.

‘The longest I've been clean (abstinent) was six months, seven months treatment down in [Town]. [I] stayed clean for another couple of months, and it was fine... It was sort of like… an hour's extra sleep for every week I was clean… *my natural rhythms* were nothing like I was when I was a teenager… I'd get seven, eight hours sleep, seven, eight, wouldn't need any more than that. So I would be awake, yeah. I'd be awake, probably after six months I would be awake at eight o'clock, nine o'clock, depending what time I went to bed. Like normal people, I suppose.’ (Andrew C1:025)

Andrew presumes that his biography of drug use altered his ‘natural rhythms’ and with time these could be restored to being like ‘normal’ people. We see here a grasp of the bundles of rhythms that make up the contingencies of sleep waves: biographical, temporal, and visceral.

Betty recalls that her childhood was characterised by a tight routine, including being sent to bed at a certain time. But sleep only came when she was convinced that her violent mother had also fallen asleep. The routine itself did not create sleep and belied the hidden rhythms associated with the socio-emotional relations of the household. Betty felt that once she was asleep it was of good quality, in part because she worked hard (physically and mentally) during the day. Betty had also experienced periods of homelessness, a period in supported accommodation, and a period of working a 3 p.m.–11 p.m. shift (during which period she described her sleep as being ‘very good’). There had been ‘on/off periods’ of alcohol and heroin use during her life. However, following a serious sexual assault, Betty recalls that she ‘turned to heroin’ and then to drink and ‘just really hit the fuck-it button hard’. Now, sleep in residential treatment did not come easy:

‘When I got into heroin when I was 21, in fact the weed as well, the weed used to make me all mongy. But then your tolerance levels build up, and if anything, if you start off smoking like a fiver's worth a day, that will make you knackered and that'll make you sleep obviously all the time. But then after X amount of time, that wears off and your body is almost looking, this is my opinion, your body is looking for that sleep again or that sedation, and when it doesn't get it, it almost makes it more alert, more kind of looking for it. It sounds ironic, but then it's that physical craving for it. You just need more and more of it and the heroin is the same.’ (Betty C2:018)

**Fig. 4:**
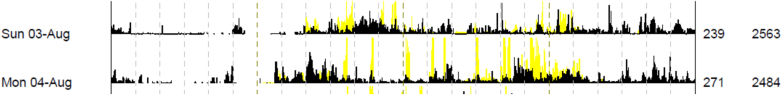
Snapshot of Betty.

Betty emphasises that: ‘when you're in addiction, it's almost like the drugs have taken over your physical self, and there is only one solution, well, there's two, there's get clean and *learn to sleep properly or use something to sleep*’. According to Betty, ‘when mental stress and not addiction has affected my sleep’, the thing that ‘works’ is smell: ‘so like whether that's an incense stick or something silly like I used to, I even now, melt chocolate in a saucer’. Drawing on the language of Schwanen et al. (2012), the smell of melted chocolate in a saucer or an incense stick can be seen as ‘smellscapes’. These provide ‘atmosphere’, but in their absence, Betty relies on ‘activity rhythms’ (Degen, 2010, Bennett, 2015) (see Fig. 4).

In talking through her actogram, which reveals much night time activity, positioning her in the top right quadrant of Fig. 2, Betty explains that:

‘I'll come downstairs and I'll have a coffee and go outside for a fag. And if someone's up, I might stay outside chatting with them for half an hour and then I'll go upstairs and kind of just read books and stuff for a little while, and then, so basically just read, self-study in my bed. I don't tend to do a lot else.’

For Betty, like Andrew, her endogenous (biological) rhythms have been broken by substance use. Without an earlier childhood association of routine and sleep to return to, and with no assurance that biological imperatives will return, Betty's *sleep waves* become determined by a reflexive need for her to ‘learn to sleep properly’; something which cannot be achieved through the enactment of routines alone.

In sum, *sleep waves* are a complex meeting of experiential and aggregate rhythms. Whilst differences between individuals exist, all participants recognised a need for individual and institutional rhythms to align. As Wolf-Meyer (2011a) argues, rather than acquiescing to the hegemony of other social actors, ‘individuals are coerced by and consent to what is discursively produced as their own biological natures’ (p.879). In other words, what constitutes their normal biological rhythms is socially constructed. Individuals, he argues, are not products of institutions, but rather ‘the lives of individuals make sense of the structure of institutions and vice versa.’ Natural hegemonies ‘become universal and inevitable by rendering individuals as of-the-same kind as institutions’ (Wolf-Meyer, 2011a: 880). These natural hegemonies bind individuals and institutions together through a shared logic. This, he argues, can be seen in American attitudes toward sleep which accept certain spatiotemporal rhythms as natural for both individuals and institutions. For Orla, Jeff and Tina, the spatiotemporal rhythms of (natural) sleep and the institution of rehabilitation have become similarly synonymous. For those who were not quite at that point (yet), experiential and aggregate rhythms remained separated but with an aspiration for a future alignment. As Andrew says, ‘if I want to hold down some kind of fucking life…’. The question of ‘alignment’ therefore merits further consideration.

## 7 Alignment and ‘ritualised routines’ in liminal spaces

The above discussion highlights how the sleep/wake routines of residential rehabilitation are desired and seen as ‘natural’ by residents and institution alike. The routines and structures also take on a further purpose. We cannot directly will sleep but we can, as Crossley (2004) suggests, ‘call on sleep’ by way of ritual, or ‘body techniques whose principle purposes concern the manipulation of our individual psychological and our social states, our subjective and intersubjective being’ (Crossley, 2004:46; Williams and Crossley, 2008). Such rituals may include the imitation of sleep; laying down in bed, ‘on my left side, with my knees drawn up’ (Merleau-Ponty, 1962: 163). Residential rehabilitation seeks to ‘call on sleep’ through the imposition of routine and structure seen as ‘natural’ by both individual and institution. In highlighting the importance of routine in their earlier lives, Orla, Jeff and Tina suggest that they had once acquired the embodied know-how or ‘acquired the particular ritual in question as a body technique and . . . [were] thus disposed to respond to the initiation of the ritual as a call to order’ (Crossley, 2004: 46). This helped to synchronise their sleep waves. For these residents, the ritual call to order had always been closely linked to routine; framing the experience of sleep and in turn shaping the belief (Crossley, 2004:44). Sleep becomes structure.

Routine is presumed to be rooted in biology and becomes both the way sleep *should be* and a way to *achieve* sleep. Calling on sleep through ‘ritualised’ routine, however, is complex. Even those who have embodied know-how also need the capacity to channel and use specific body-techniques. *Sleep waves* are more than the relationship between the current demands of treatment and embodied, visceral memories; they also encompass spatiotemporally immediate and extended influences – things that happen within and beyond the resident's bedroom. Orla's eurythmia, for example, exists in folded polyrhymic relation to her roommate's lack of chit chat and noise, with the dressage of the daytime routine there to create sleep, and helped by the slowed down rhythm of the (noiseless) night. At the same time as stressing the importance of routine, Jeff suggests a ‘bending’ of the body because he has to take others into account; there are people who ‘*need* more sleep than him’, most notably the ‘drug addicts who have the real *problem*’ (as opposed to his addiction to alcohol). This, then, requires complex spatiotemporal coordination to the rhythms and pace of others and ‘an added layer of discipline’ (Jeff C1:008).

*But there is some sort of…. there is an added layer of discipline.* If you are thinking well it is 50/50 on whether I turn the light on or read, if I was in the [other place]I would definitely read because I am not going to disturb anyone else. You have got the added factor of, “god, I might wake up the person next door” and that's going to…well that's number one going to interrupt his sleep and his waking up might hinder my sleep later so it's a triple whammy (Jeff 008)

Embodied emotions also impact *sleep waves*. Tina, who as we saw above, reports the benefits of her imposed structured routines in childhood, nevertheless finds that dreams affect her sleep. Whilst routines may act as a ‘ritualised’ way to induce sleep and affect Tina's intentional life, they cannot necessarily fend off or circumvent negative emotional states (cf. Crossley, 2004: 43).

‘I am definitely an emotional person, so I get quite anxious about things… But being here is quite nice because it is structured, so I know what I have got to do next... but that is just general living. Things that have gone on in the past and things that are going to happen in the future play on my mind a hell of a lot and I think it does disrupt my sleep to the point where I have them vivid dreams. But I don't necessarily…. I can't relate them to reality, I can't relate my dreams to reality.’ (Tina: C1:004)

Our participants' sleep is also embedded within spatiotemporal networks. Some residents experience the residential setting as cloistered; highly differentiated from the immediate and extended and inside and outside time and space. Its markers and pacemakers are experienced as: ‘safe’ ‘structured’, ‘slowed down’, not the ‘real world’. But the ‘real world’ does seep through. Deidre's *sleep waves* are clearly influenced by the coming together of inside and outside time spaces. Deidre, who is currently going through a divorce, finds biographical, experiential, temporal and spatial rhythms intermingle.

‘I'm exhausted. I mean I've got text messages, phone calls, solicitors, emails, blah de blah de blah, and this programme is heavy. This programme is really, really heavy… And in the evenings we carry on because we have to do meetings. And like last night, I did the programme all day, I had to speak to the counsellor on the phone, total nightmare, then I had a two-hour appointment with a solicitor, then I went to a fellowship meeting. I got back here just after 10 o'clock last night, had been up six, quarter to seven, and then by the time I'd sorted out things here that I needed to do to prepare for today, it was certainly, I don't know, I like rush to get the light out, because I think oh my god, then working out how many hours sleep I'm going to get before the alarm goes off, rushing.’ (Deidre C2:035)

For Deidre, the ‘marker’ of divorce is having an ‘accordion effect’ – creating ripples – but within residential treatment these ripples have to play out against rules and routines which are normally absent from adult life: to borrow from Collins, there is too much deliberation and self-consciousness which removes a natural flow (Collins, 2004: 53).

Even where routines have successfully been enacted as a ‘call to sleep’, the liminal nature of the residential setting continues to impact on *sleep waves*. This manifests itself in significant ways as illustrated in Peter's accounts. The anticipated transition from ‘the bubble’ of residential treatment to the ‘outside world’ caused anxieties to surface. Peter, who was moving towards the end of his stay, illustrates how the strong pacemakers of residential treatment have become a ‘call to sleep’, meaning that he now experiences sleep as *caused* by, and synonymous with, these entraining entities. This leads to the perception that future sleep can only be achieved through the continued replication of residential time-space.

‘I don't imagine myself getting no sleep at all (once treatment is over). But because of the nature and structure of [the named treatment programme], I think as long as I am in that structure, I'll by and large think, sleep will be okay. When I do leave and that'll involve certainly lots of recovery support networks, some type of work, something that I have to be up early for. Because having that structure and having to be up early is the biggest way I am likely to make sure I will have, or maintain, a good sleep pattern, even if it is not the best sleep I could possibly get.’ (Peter C1:003)

Peter is adamant that he is not going to leave treatment ‘until I know that I have got structure to walk into’. His account points to the challenges of situating specific spatiotemporal routines as both the way sleep *should be* and a way to *achieve sleep*. The naturalising discourse that underpins the institution's routines and rhythms becomes a source of anxiety which can work back to influence *sleep waves*.

## 8 Concluding discussion

In this article we have drawn on Lefebvre's rhythmanalysis as a ‘way in’ to explore sleep within residential treatment centres. This approach encourages consideration of the endogenous and exogenous, biological and social dimensions of sleep. As Lefebvre (2004: 80) states, ‘the hypothesis of rhythmanalysis’ presumes that the body ‘consists of a bundle of rhythms’ such as ‘homeostatic’, ‘cybernetic’, ‘cosmic’ and ‘social’ rhythms that are ‘in a perpetual interaction’. Rhythm, he argues, conceptually ‘reunites quantitative’ with ‘qualitative aspects and elements’ of life.

‘Rhythm appears as regulated time, governed by rational laws, but in contact with what is least rational in human being: the lived, carnal the body. Rational, numerical, quantitative and qualitative rhythms superimpose themselves on the multiple natural rhythms of the body (respiration of the heart, hunger, thirst etc), though not without changing them’ (Lefebvre, 2004: 9).

Throughout this paper we have undertaken an ‘analytic operation that consists of opening and unwrapping the bundle’ (2004:9) of rhythms. Sleep, like waves, have multiple rhythms that come in succession but are uniquely shaped by spatial, temporal, biological configurations.

*Sleep waves*, as an analytic, enables us to actively capture and explore the polyrhythmia of every day/night life, encouraging us to look at spaces, routines, rituals, movements and bodies, as both repetitious yet unique. This is not necessarily alien to disciplines such as chronobiology or chronogeography. We find on the contrary that there is a shared grammar of ‘rhythm’ across these disciplines; yet as noted above they have tended to prioritise *either* endogenous (internal) *or* exogenous (external) rhythms. Within the current study, the mix of actigraphy and interviews allowed us to plot movements across time and space whilst also allowing these movements to be subjectively interpreted by participants themselves. We have not, however, included data or analysis of endogenous processes such as homeostatic processes, cortisol rhythms and so on. This was beyond the scope of our study. What our conceptual approach indicates is that there is potential for interdisciplinarity to do so and this could produce a rounded appreciation of *sleep waves*.

Studying *sleep waves* does not entail the uncritical adoption of a bio-medical view of sleep. Shifting to a focus on waves already moves us from an understanding of sleep as simply being other than awake, and recognises sleep to be multifaceted. Nor need a focus on sleep waves privilege any particular ontology of sleep, but instead allows for a closer dialogue to stitch together various sleep ontologies (Nettleton et al., 2017, forthcoming). Nevertheless, exploring *sleep waves* in an interdisciplinary way will invariably lead to troublesome questions for all disciplines. The current findings suggest that (i) residential rehabilitation is built around routines and structures which mirror those found in institutions throughout wider society; (ii) there is an individual desire to align to these routines and structures as the ‘correct way to sleep’; and (iii) routines and structures become seen as the way to ‘achieve sleep’. Sleep is therefore both ‘desired’ and ‘called on’ through the lens of routine. This double existence presents a range of challenges for both those who cannot approximate the desired forms and those who do. This is not a normative statement, but the findings do prompt questions about the presumed ‘natural’ day/night cycles and ways of sleeping. Similar questions are permeating ‘sleep science in subtle ways’ (Wolf-Meyer, 2011b:967). For example, in other institutional settings sleep science has informed or at least been deployed to discuss strategies to personalise work and school schedules to individual chronotypes (Wittmann et al., 2006) and to genetically identify and assign people to jobs with appropriate shift schedules (see Wolf-Meyer, 2011a). We can therefore see that there is scope for our current findings to engage with and invite further dialogue with ‘sleep scientists’ as to what ‘natural’ *sleep waves* might look like. Our own stance within these discussions is that whilst there may be ‘natural’ circadian rhythms, these are so overlaid with the social that there is no possibility of separately discerning them (Cf. Meadows, 2012). *Sleep waves* are inherently polyrhythmic and the alignment of these multiple spatiotemporal and embodied rhythms is a fragile ongoing process; consequently, our appreciation of them must be genuinely interdisciplinary.
